# Bone as a Biomarker of Ageing and Senescence: Connecting Musculoskeletal Health to Longevity via Diet, Nutrition and Exercise

**DOI:** 10.3390/biology15161377

**Published:** 2026-08-12

**Authors:** Hannah Beaumont, Sam Guest, Shelly Pathak, Yashaswini Premjit, Elena A. Jones, Payal Ganguly

**Affiliations:** 1 STILL NOT DONE, Otley, Leeds LS21 3NX, UK; hannah@wearestill.co.uk (H.B.); sam@wearestill.co.uk (S.G.); 2 Novogene Europe, 25 Science Park, Milton, Cambridge CB4 0FW, UK; shelly.pathak@novogene-europe.com; 3 School of Food Science and Nutrition, University of Leeds, Leeds LS2 9JT, UK; y.premjit@leeds.ac.uk; 4 Leeds Institute of Rheumatic and Musculoskeletal Medicine, Faculty of Medicine and Health, University of Leeds, Leeds LS7 4SA, UK; 5 Leeds Biomedical Research Centre, Leeds Teaching Hospitals NHS Trust, Leeds LS7 4SA, UK

**Keywords:** longevity, bone ageing, biomarker of ageing, senescence, healthy ageing, musculoskeletal (MSK) health, bone marrow stem cells, anti-ageing strategies, interventions

## Abstract

Advancing age is often accompanied by a gradual decline in musculoskeletal (MSK) health and integrity, making the skeleton not just a structural framework; but a dynamic indicator of biological ageing. Bone tissue undergoes continuous remodelling through tightly regulated interactions among osteocytes, osteoblasts, osteoclasts, progenitor cells or mesenchymal stem/stromal cells (MSCs) and growth factors. With advancing age, this balance shifts toward increased resorption, increased adipose tissue formation, reduced bone formation, and deterioration of bone microarchitecture. This eventually can lead to frailty, increased fracture risk and other age-related diseases (ARDs) due to ageing, cellular senescence and inflammaging. Interestingly, bone-derived changes often precede clinical manifestations of these ARDs, suggesting its potential use as an early biomarker for biological ageing and longevity risks. In our review, we explore the possibility of using the bone as a biomarker for ageing, followed by anti-ageing strategies to encourage bone longevity. We begin by discussing our current knowledge of the bone, the bone marrow (BM), changes in the BM with ageing and current methods of tracking bone ageing; both clinically and experimentally. We then explore studies that investigated the anti-ageing strategies, specifically focused on diet, exercises and lifestyle to shift towards enhanced bone health and longevity. Finally, we discuss the limitations of achieving this, what the future of this field may look like and what methods may be used to translate the idea of bone as a biomarker of ageing, from bench to bed.

## Simple Summary

As we age, our bones and muscles gradually become weaker. Bones are living tissues that constantly renew themselves through a balance of old bone being removed and new bone being formed. With age, this balance shifts: more bone is lost, less new bone is made, fat builds up in the bone marrow, and bone structure becomes weaker. These changes can lead to frailty, fractures, and other age-related diseases (ARDs). As bone health often declines before symptoms of ARDs appear, bones may serve as an early warning sign of biological ageing. This review explores bone ageing, ways to measure it, and lifestyle strategies such as diet and exercise to help maintain stronger, healthier bones for longer time.

## 1. Introduction

Ageing and age-related changes are a combination of complex natural processes that are often associated with several co-morbidities and age-related diseases (ARDs). By 2050, the world population will have nearly 22% people over the age of 60 [1]. Advancing age by itself is not a problem as several individuals/regions with people over the age of 60 are known to lead a fit and healthy life [2]. Among the ARDs, specifically musculoskeletal (MSK) diseases like osteoarthritis (OA), osteoporosis (OP), rheumatoid arthritis (RA) and others, reduce the mobility of the individuals due to pain and stiffness, severely impacting the quality of life (QOL). While these diseases have established a fundamentally distinct pathogenesis, alterations in the bone, inflammation and cellular senescence play key roles in all these diseases [3,4,5,6]. Thus, OA, OP, and RA share mechanisms including chronic inflammation mediated by tumour necrosis factor alpha (TNF-α), interleukin one beta (IL-1β), and interleukin six (IL-6), dysregulated bone remodelling through the RANK/RANKL/OPG axis, cellular senescence, as well as immune skeletal interactions. MSK diseases are also known to be amongst the largest contributors towards health and economic challenges globally [7].

Bones form our skeletal system, providing support, structure and protection to internal organs. Bone marrow (BM) within the bones is the site of haematopoiesis or blood formation and is a niche environment for stem cells, immune cells, cytokines and growth factors [8]. It is a dynamic organ forming the core of the MSK system. The stem cells involve haematopoietic stem cells (HSCs) that eventually form into different types of immune cells [9]; and mesenchymal stem/stromal cells (MSCs) that are progenitors of bone, cartilage and fat. MSCs play a critical role in bone formation, bone healing, repair and regeneration [10,11]. With cells beginning their journey within the BM, tracking the bone and its cellular components with advancing age has provided us with immense knowledge about these age-related changes so far.

We know that there is a decline in number of MSCs with advancing age [12,13], a bias towards adipogenic over osteogenic differentiation, making the BM ‘fatty’ as we grow older [14,15]. We also know that there is ‘myeloid skewing’ with ageing, indicating that HSCs tend to shift their differentiation towards myeloid cells (granulocytes, monocytes, erythrocytes and thrombocytes) instead of maintaining the stability between lymphoid (T cells, B cells, NK cells) and myeloid cells [16,17,18]. These age-related changes can be explained by several ‘hallmarks of ageing’ [19,20] including cellular senescence, which is the stage where a cell has permanently lost its ability to proliferate due to accumulation of damage over the years [21,22,23].

Senescent cells produce inflammatory cytokines and factors, further adding to the damage, and this is known as senescence-associated secretory phenotype (SASP) [24,25]. Senescence and SASP have also been associated with accumulation of chronic low-grade inflammation with advancing age, referred to as ‘inflammaging’ [26,27]. Both cellular senescence and inflammaging are known to contribute towards MSK ARDs [6,28,29]. Thus, tracking these intricate changes at the cellular level will further provide us with invaluable information to tap into strategies for healthy ageing and bring MSK longevity within reach. Current methods of tracking bone ageing include imaging methods (radiography, Dual-energy X-ray Absorptiometry (DXA) for bone mineral density (BMD), and high-resolution peripheral QCT (HR-pQCT) for trabecular microarchitecture, histological methods using bone biopsies and measuring senescence in bone cells and progenitors using senescence-associated beta-galactosidase (SA-β-gal) assay [30,31].

While several techniques are used to study these age-related changes, understanding how diet, nutrition and exercise impact bone health, specifically at the cellular and genetic level, is critical to combat MSK ageing and senescence. Diet, exercise, social life and overall one’s lifestyle is well reported to impact MSK health [32,33]. This article aims at highlighting these key connections between the effects of diet, nutrition and lifestyle with bone health and MSK ageing.

We begin by discussing the changes in the ageing bone, its genetics and the methods used to track these age-related changes. We then initiate the idea that the bone may be utilised as a biomarker of ageing. This is followed by outlining evidence of anti-ageing strategies with a focus on diet, nutrition, exercise and lifestyle on MSK ageing and bone health. We dissect this information through an interventional perspective and note that these can be potentially used for future prevention of MSK diseases and bring bone longevity within reach. Our aim is to equally shed light on the concept of bone as a biomarker of ageing, as well as the evidence of the effect of diet, nutrition and lifestyle on MSK health, treating these two topics as co-primary aims. We summarise the challenges and future directions of the field, outlining what the next steps can be to translate the idea of using the bone as a biomarker, from bench to the bedside. We hope that the contents of this article, including multi-disciplinary approach to maintain QOL in patients with MSK ARDs and anti-ageing interventions, provide a reason for different scientific, research, medical and industry professionals to collaborate in this ever-exciting field of bone ageing.

## 2. Methodology and Search Strategy

We started with the key words of ‘bone ageing’, ‘biomarkers’, ‘cellular senescence’, ‘diet’, ‘exercise’ and ‘anti-ageing strategies’ as our literature search for our narrative style review; in which the criteria for a systematic review do not apply. These were further narrowed down by adding ‘musculoskeletal’ to the search to eliminate focus from other systems. Both PubMed and Google scholar were used as platforms for extraction of information on these specific areas of research. Further, we included full texts and focused on literature within the last two decades. Review articles, research articles, and editorials primarily focusing on bone ageing were included. Publications that did not have full text were excluded. Quality of publications used was checked by the authors writing this manuscript (HB, SG, SP, YP, EJ and PG). FigureLabs.AI (https://www.figurelabs.ai/, (accessed on 20 June 2026)), a web-based scientific illustration platform (free version) was used to generate Figure 3. We acknowledge that despite our efforts of conducting a comprehensive literature search, the risk of bias cannot be ignored. The strategy we employed for our manuscript, using the keywords, inclusion/exclusion criteria and our interpretation of the information to prepare our article, has possibly overlooked some of the studies in the field. The manuscript was written with the resources available to us during manuscript preparation, and we do not claim to be exhaustive.

## 3. The Bone and Bone Marrow (BM) in Health and Ageing

### 3.1. Age-Related Changes in Bone and Bone Diseases

The BM in the bone is central to structural and functional integrity of the bones and the MSK system, including the formation of progenitor cells, differentiation to other cell types, bone formation and bone resorption, also known as bone remodelling. It is an essential process ensuring bone competence that occurs throughout a person’s lifetime consisting of bone resorption by bone eating cells or ‘osteoclasts’ followed by the formation of new bone by ‘osteoblasts’ [34]. This process is performed due to the interaction between receptor activator of nuclear kappa-β (RANK)/RANK-ligand (RANKL)/osteoprotogerin (OPG) system [35]. A key role is played by osteocytes here in mechano-sensing, lacuno-canalicular communication (LCN) and regulation of bone remodelling.

Osteocytes are the principal mechano-sensory cells of bone and play a central role in skeletal ageing. Embedded within the mineralised matrix, osteocytes detect mechanical strain and transmit signals through the extensive LCN, coordinating communication with osteoblasts and osteoclasts to regulate bone remodelling [36]. Ageing is associated with osteocyte loss, cellular senescence, reduced dendritic connectivity, and degeneration of the LCN, resulting in impaired mechano-sensing and a diminished anabolic response to physical loading. Increased production of sclerostin (SOST) and pro-inflammatory SASP factors further suppresses Wnt/β-catenin signalling and osteoblast activity while promoting bone resorption [37]. Consequently, osteocyte dysfunction contributes to bone fragility, cortical porosity, and impaired adaptation to exercise, making osteocytes and their signalling pathways attractive biomarkers and therapeutic targets for skeletal ageing and OP [38].

**Figure 1:**
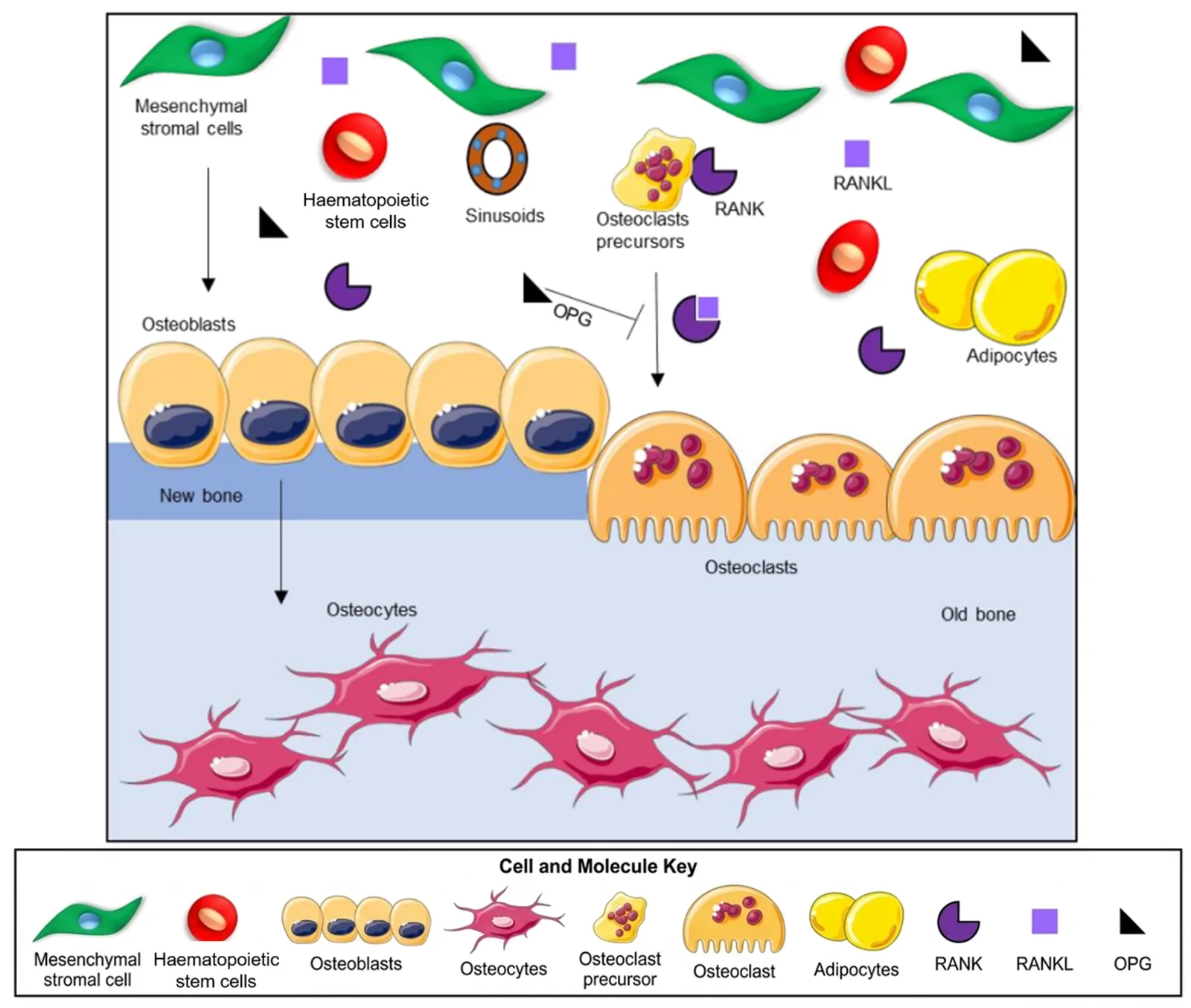
Key cells and molecules involved in bone remodelling (as presented in corresponding author’s thesis [13]).

RANKL is a member of the TNF superfamily produced by MSCs, osteoblasts and osteocytes, and acts a driver for bone resorption. RANKL binds to the RANK receptors on the osteoclast precursors and activates the resorption of the bone by osteoclasts for their survival. OPG is a soluble decoy for RANK produced by MSCs and osteoblasts and binds to RANKL to prevent the biding of RANK to RANKL, thus stopping the activation of osteoclasts and bone resorption [39,40]. OPG sequesters RANKL, halts the activation and maturation of osteoclasts and protects the bone from any further resorption (Figure 1). The balance between bone formation and resorption is essential for maintaining the quality of bone; however, this balance steeps towards increased bone resorption over bone formation as we get older, contributing to age-related bone loss. Several other changes occur in the BM with age and these are outlined in the sections below.

#### 3.1.1. Marrow Adiposity

**Figure 2:**
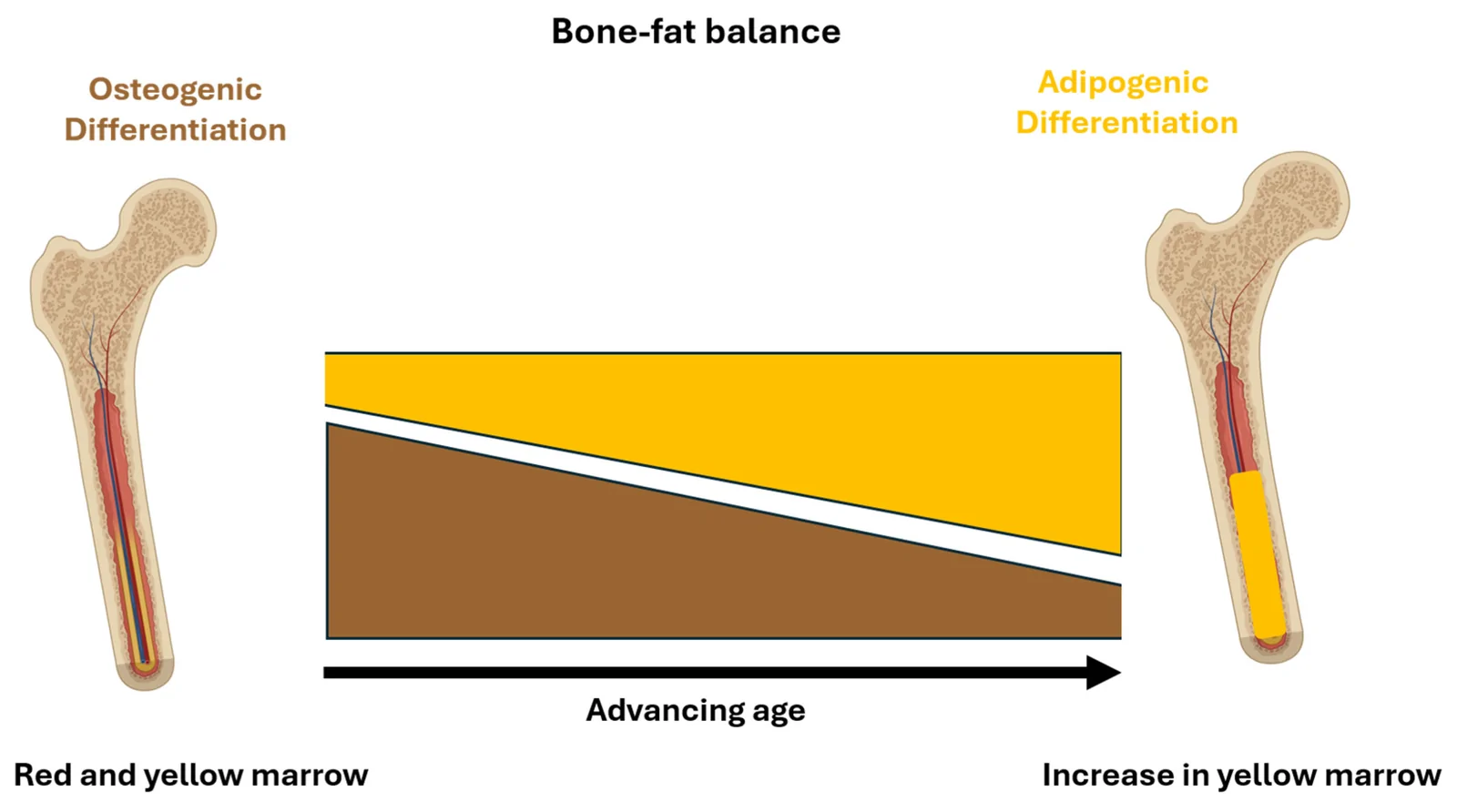
Bone-fat balance across advancing age.

Overall, the marrow’s adiposity increases with advancing age with higher levels of yellow marrow evidenced over red marrow region. This can be accredited to the shift in BM MSCs in their differentiation ability, skewing towards adipogenic, more than osteogenic differentiation with advancing age [41]. BM-MSCs are trilineage in their differentiation capacity, which means that they may differentiate into bone, fat or cartilage. While moderate level of BM adipose tissue (BMAT) is a sign of a healthy marrow, increasing levels of BMAT indicates a shift towards more fat formation at the cost of bone formation [42,43]. This shift is often observed in older donor cells, wherein, the MSCs skew toward adipogenic differentiation, disrupting the bone–fat balance (Figure 2).

Within the BM, the site where BM-MSCs are formed is located between bone trabeculae of cancellous (spongy) bone of either axial or peripheral skeleton. It is phenotypically classified as either red marrow, mainly filled with haematopoietic cells, or yellow marrow, characterised by increased formation of adipocytes [44]. Several cells and molecules play a key role in this shift.

RANKL is secreted by bone and fat progenitors (MSCs) within BM and studies have reported that increased RANKL from BM adipogenic precursors, promote osteoclastogenesis during bone remodelling, contributing to marrow adiposity [45,46,47] and to diseases like OP [48,49,50]. Additionally, the BM-MSCs that differentiate into adipocytes or fat, secrete adipokines like adiponectin and leptin. While these play an important role in maintaining bone homeostasis, an age-related increase in their production further contributes to the loss of bone–fat balance within the BM. Apart from these key players, a number of other factors play a role in increased marrow adiposity with advancing age. For example, transcription factors like Peroxisome Proliferator-Activated Receptor gamma (PPAR-γ) are the most important drivers of adipocyte differentiation and its deletion has shown to protect age-related bone loss in mice [51]. Other factors, including transcription factors responsible for osteogenic differentiation like RUNX2 and osterix decline with ageing and contribute to marrow adiposity [51,52].

#### 3.1.2. Changes in Stem Cell Numbers and Functions

The two stem cell populations in the BM, i.e., MSCs and HSCs, undergo different changes in their numbers across lifetime. In vitro and ex vivo investigations from our previous studies [53,54] and other research groups [55,56] have indicated a decline in the number of BM-MSCs with advancing age. Interestingly, a steeper decline is observed in the fifth decade of life between 40 and 60 years of age, which then has been reported to stabilise after reaching the sixties [53]. Reduction of BM-MSCs and decline in their functional capacities with advancing age, has been identified as ‘stem cells exhaustion’ among the hallmarks of ageing and can be attributed to cellular senescence [57,58]. Reduction in the number of BM-MSCs indicates a decline in the number of MSCs available within the BM to differentiate in bone, fat and cartilage and has an impact on the overall demarcation of MSCs towards their selected fate and functional capacities.

These functions include proliferation and colony formation, differentiation, migration, supporting haematopoiesis and immunomodulatory activities. Studies have indicated a decline in each of these functions of MSCs with advancing age, and a summary of studies focusing on human derived cells from the last five years indicating these have been indicated in Table 1 below. While the decline in each of these functions can be correlated to the decline in their numbers, the exact mechanisms are yet to be solidified.

HSCs, on the other hand, are a unique case that have been reported to significantly increase in numbers with advancing age. However, this is thought to be compensatory due to the cumulative decline in several other functions associated with HSCs [62]. Proliferation capacity, differentiation abilities, regeneration competence and other functions have been reported to be compromised with advancing age, despite the increase in their numbers (Table 1). Much like our knowledge of age-related changes within MSCs, we are aware of the hallmarks of HSC ageing that are similar to the hallmarks of MSC ageing; however, their exact mechanisms are still being explored by scientists globally [63]. There are various techniques that have been used to measure and quantify these age-related changes in these stem cells within the BM, outlined below.

### 3.2. Current Methods to Track Age-Related Changes in the Bone

#### 3.2.1. Diagnostic Tools and Clinical Methods

Imaging techniques provide a non-invasive, longitudinal assessment of bone ageing in vivo. DXA measures BMD, widely used for fracture risk prediction but is limited in providing high resolution bone microstructure. High-resolution peripheral quantitative computed tomography (HR-pQCT) offers three-dimensional (3D) evaluation of trabecular and cortical architecture, including porosity and thickness, allowing detection of subtle age-related deterioration [64]. Clinically, other tests like fracture risk assessment tool (FRAX) use a combination of clinical risks and DXA to predict the fracture probability over a decade in a person [65]. Trabecular bone score (TBS), which is also derived from DXA after analysis of the grey-level variation on the scan, is emerging as an indirect indicator of bone microarchitecture [66].

Bone turnover markers (BTMs) analysed from blood or urine samples provide a short-term snapshot of bone health and usually include the measurement of P1NP (Procollagen Type 1 N-Terminal Propeptide) for bone formation and C-terminal telopeptide (CTX) for bone resorption [67]. Emerging molecular imaging approaches, such as targeted probes for senescence markers, enable visualisation of senescent cell burden and spatial distribution within bone tissues. Additional artificial intelligence (AI) tools will further enable extracting information towards age-related changes in the bone [68,69]. Together, these modalities integrate structural and cellular insights, improving the ability to track bone ageing progression and responses to interventions.

#### 3.2.2. Experimental Approaches

BM-MSCs morphology under the microscope, even without any other kind of staining, can predict its senescent nature with elongated appearance, disproportionately larger nucleus and increased granularity [70]. Senescent MSCs can be further identified using techniques involving metabolic studies, reactive oxygen species (ROS) and protein arrays [71]. A study in 2023 compared young and old BM-MSCs and found increased SA-β-gal stained cells, ROS and expression of inflammation markers like IL-6 and Interleukin-8 (IL-8) in MSCs from older donors when compared to younger donors [52]. Other commonly used techniques include functional assays to evaluate proliferation capacity (EdU/Ki67), telomere shortening and differentiation potential. Flow cytometry and autofluorescence methods also enable quantitative analysis of senescent cells.

Common techniques to measure HSC ageing and senescence also integrate several functional assays. One of them includes molecular profiling by tracking clusterin [72]. In vitro, it is assessed using SA-β-gal staining, p16^INK4a^/p21 expression and DNA damage markers within clusters of cell populations in HSCs [73]. In vivo, gold-standard approaches include transplantation assays, which assess long-term repopulation and regenerative capacity, and lineage tracing to track clonal dynamics [74,75]. Advanced single-cell sequencing and epigenetic profiling reveal ageing-associated transcriptional and clonal changes in HSC populations.

Multi-omics approaches can provide high-resolution insight into BM ageing by integrating data across different layers. Transcriptomics (bulk and single-cell RNA sequencing) can identify gene expression changes associated with senescence, including upregulation of cell-cycle inhibitors and inflammatory SASP components. Importantly, single-cell sequencing technologies uncover cellular heterogeneity within BM, distinguishing aged subpopulations of MSCs, HSCs, immune cells, and mapping lineage-specific ageing trajectories [76]. Spatial and multi-omics integration can further enable linking molecular signatures to niche organisation and function. Together, these approaches have the potential to generate comprehensive ageing signatures, improving mechanistic understanding and enabling precise identification of SASP pathways and therapeutic targets.

Considering the bone itself provides number of indicators for ageing, along with its several components including the BM stem cells; it is worth taking into account that tracking the bone along its lifetime will provide us with details needed to dissect senescence and cellular ageing. With the recent discussions around biomarkers of ageing across the globe, it is imperative that we evaluate the bone, its niche, its cells and its genetics to amplify the potential of using bone as a biomarker of ageing.

### 3.3. Genetics of Bone Ageing and Senescence

Genetic, epigenetic, and cellular factors interact intricately to control bone ageing and senescence. The balance between bone formation and resorption, stem cell function, and the build-up of senescent cells that negatively impact the bone microenvironment are all influenced by genetic factors. The translational promise of genetics in the fight against age-related bone ailments is shown by the ongoing discovery of new therapeutic targets [76]. Changes in osteoblasts, osteoclasts, skeletal stem/progenitor cells, and the buildup of senescent cells in the bone microenvironment are essential to this process [77,78].

Bone ageing is typified by cellular senescence, which is characterised by irreversible cell-cycle arrest and a pro-inflammatory SASP that upsets bone homeostasis, resulting in decreased bone production and increased resorption [77]. Telomere dysfunction, activation of p16^INK4a^/p21^CIP1^ pathways, altered cell-cycle-related gene expression, and dysregulation of important pathways like Wingless/Integrated (Wnt) signalling are all genetically linked to bone ageing. Recent research has linked MEN1-mTORC1 signalling to osteoblast senescence [79,80,81].

Due in part to the activation of senescence pathways including CDKN2A (p16^INK4a^) and CDKN1A (p21^CIP1^), genetic and cellular alterations linked to bone ageing result in decreased bone matrix synthesis and altered osteoblast function [76]. Additionally, mechano-sensing and LCN communication are disturbed in ageing osteocytes, which weakens bone architecture and its ability to adjust to changing mechanical strain [82]. Concurrently, the balance is shifted toward bone loss and increased marrow adiposity due to deregulation of bone remodelling pathways, such as decreased Wnt/β-catenin signalling (Low-density lipoprotein receptor-related protein 5 (LRP5), SOST and increased mTORC1 activity [83,84]. These discoveries have significant clinical ramifications because they point to therapeutic options like senolytic tactics, that target cells that express CDKN2A, Wnt and mTORC1 signalling modulation to increase osteoblast activity, and novel epigenetic or microRNA-based methods to restore skeletal stem cell function and age-associated gene expression [85].

### 3.4. Current Biomarkers Used and Key Pathways Involved

Bone ageing is thus currently being characterised by reduced bone mass, deterioration of bone microarchitecture, increased cortical porosity, and impaired bone remodelling. Current clinical biomarkers include BMD measured by DXA, TBS for bone quality, and BTM which reflect remodelling dynamics and predict fracture risk independent of BMD. At the cellular and molecular level, bone ageing is associated with higher SA-β-gal stained cells, reduced osteoblast number and activity, osteocyte dysfunction and senescence, increased osteoclast-mediated resorption, and declining MSC osteogenic potential. Key molecular markers include SOST, RANKL, OPG, FGF23, and components of the Wnt/β-catenin pathway, which regulate bone formation and resorption.

The Wnt/β-catenin pathway thus is a major anabolic pathway for osteoblast differentiation and bone formation, but becomes suppressed with age, partly through increased SOST expression. Osteocyte dysfunction and senescence impair mechano-sensing and skeletal adaptation to loading. Key cross-regulatory pathways inflammaging and senescence have dual pathway impact [86]. This in turn increases TNF-α, IL-1, and IL-6, stimulating osteoclast activity and suppresses osteoblastogenesis. Bone ageing is also linked to vascular ageing through shared regulators including RANKL/OPG, Wnt/β-catenin, FGF23, phosphate signalling, and osteogenic transdifferentiation of vascular smooth muscle cells, forming the bone–vascular axis. In addition, age-related decline in muscle mass and strength influence bone through the bone–muscle axis, mediated by myokines, mechano-transduction, and stem-cell senescence.

Additionally, bone ageing is being linked to dysfunction of the gut–bone axis. Age-related changes in the gut microbiome (dysbiosis) can increase intestinal permeability, reduce production of beneficial metabolites such as short-chain fatty acids (SCFAs), and promote inflammaging. These changes alter osteoblast–osteoclast balance, favouring bone resorption and contributing to osteopenia and OP [87]. The gut microbiota also influences calcium absorption, vitamin D signalling, oestrogen metabolism, and immune pathways (including the Th17/Treg balance), all of which regulate bone remodelling [88]. Consequently, age-related gut dysbiosis may accelerate skeletal ageing, while microbiome-targeted interventions (diet, prebiotics, probiotics) have emerged as potential strategies to preserve bone health. Table 2 below summarises these markers.

## 4. Strategies for Bone Longevity

### 4.1. Introduction to Anti-Ageing Strategies: The Three-Pronged Approaches

**Figure 3:**
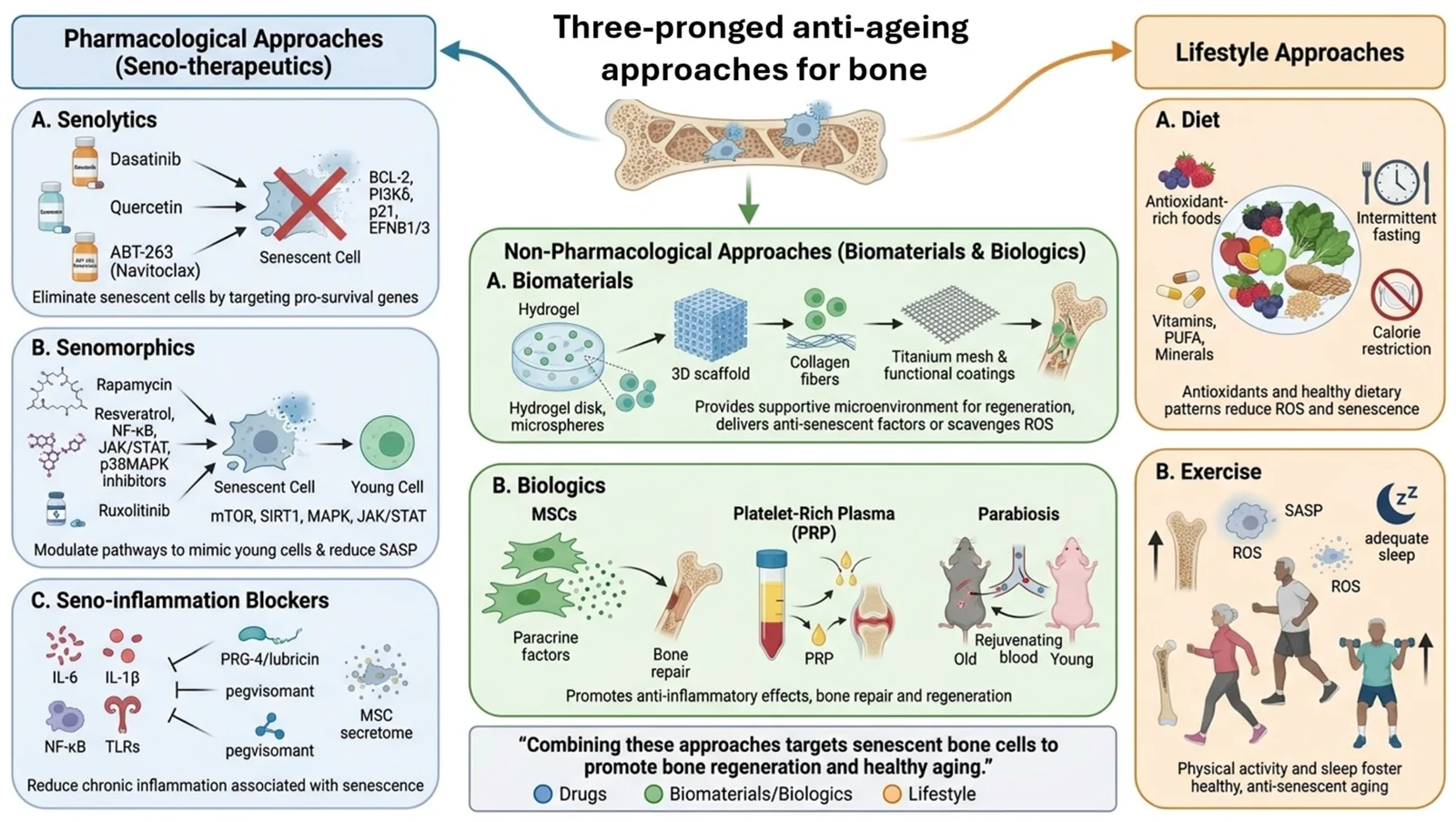
Three-pronged approaches to anti-ageing strategies [6] (generated by FigureLabs.AI).

There are several anti-ageing strategies that are being employed to help regenerate bone and provide it with a longer health-span or longevity. These approaches may be broadly classified as (a) pharmaceutical, using drugs and medication, (b) non-pharmaceutical, using biologics like platelet-rich plasma (PRP), and regenerative biomaterials like shell nacre cement (SNC), (c) using diet, nutrition and lifestyle [6] (Figure 3).

Pharmaceutical strategies aim to target the cellular and molecular drivers of bone ageing and are among key emerging strategies. Anti-resorptive drugs such as bisphosphonates and denosumab reduce osteoclast activity, preserving bone mass [57]. Anabolic agents, including teriparatide (PTH analogues) and romosozumab (sclerostin inhibitor), stimulate osteoblast function and bone formation. Evolving anti-ageing approaches include senolytics (e.g., dasatinib and quercetin), which selectively remove senescent cells, and senomorphics, which suppress SASP. Modulating pathways such as Wnt/β-catenin, mTOR, and AMPK are also being explored to restore MSC osteogenic potential and reduce marrow adiposity.

Biological therapies focus on enhancing the regenerative microenvironment. Platelet-rich plasma (PRP) provides growth factors (e.g., PDGF, TGF-β, VEGF) that promote osteogenesis and angiogenesis. Stem cell-based therapies, particularly using MSCs, aim to replenish osteogenic capacity and modulate inflammation. Biomaterials such as scaffolds (collagen, hydroxyapatite, bioactive ceramics) provide structural support and mimic the bone extracellular matrix, facilitating cell attachment and differentiation. Advanced approaches include smart biomaterials, regenerative materials and controlled drug delivery systems, which can release osteogenic factors in a targeted manner, enhancing bone regeneration in ageing tissues. The effects of microenvironment (serum or extracellular matrix (ECM) from young donors) have been evidenced to enhance proliferation and regeneration capacity of BM-MSCs from older donors [54,56].

Lifestyle interventions remain fundamental to maintaining bone health. Adequate intake of calcium, vitamin D, and protein supports bone mineralisation and muscle function. Nutrients with anti-ageing properties, such as omega-3 fatty acids, antioxidants, and polyphenols, help reduce inflammation and oxidative stress. Regular weight-bearing and resistance exercises stimulate bone formation and improve mechanical strength [89]. Additionally, avoiding smoking and excessive alcohol intake preserves bone quality. Emerging evidence also highlights the role of gut microbiota and metabolic health in bone ageing, suggesting diet-based modulation could indirectly enhance skeletal regeneration and delay age-related bone decline.

### 4.2. Effects of Diet and Nutrition on Bone Ageing, Senescence and Inflammaging

Ageing is associated with progressive changes in bone mass and microarchitecture as well as fracture resistance. These changes are increasingly linked to cellular senescence and its associated inflammatory signalling, oxidative stress and microbiota-mediated regulation of skeletal homeostasis [6,90,91]. It is particularly relevant because senescent osteogenic, osteocytic and immune cells may acquire an accompanying SASP, which can promote inflammation and disturb bone remodelling [6,90]. Consequently, diets are now being examined not only as a source of skeletal building blocks, such as calcium, vitamin D, and proteins, but also as a modifiable regulator of inflammation, oxidative stress, cellular senescence, bone remodelling, and gut microbiota-derived metabolites [91,92,93]. This broader view has encouraged investigation of whole foods, dietary patterns, bioactive compounds and probiotics, as summarised in Table 1.

Among food-based interventions, prunes (*Prunus domestica* L.) have one of the most developed evidence bases for postmenopausal bone health. Earlier clinical trials reported favourable effects of dried plum intake on BMD and bone turnover markers, while more recent studies refined the commonly tested intake range to 50–100 g/day, with 50 g/day emerging as a more feasible, long-term dosage [94,95,96]. In the 12-month prune study, 50 g/day prevented loss of total hip BMD in postmenopausal women, and subsequent analyses reported preservation of tibial cortical bone structure and estimated strength, as well as reduced pro-inflammatory cytokine secretion and circulating activated monocytes [86,97,98,99]. Resveratrol, a naturally occurring polyphenol present in foods such as grapes and berries, has also been examined because of its potential vascular, phytoestrogenic and anti-inflammatory actions. In the RESHAW trial, supplementation with 75 mg resveratrol twice daily improved lumbar spine and femoral neck BMD and reduced CTX, with subanalysis suggesting greater bone-protective benefit among participants also taking vitamin D plus calcium [100].

A trial with Vitamin D and Omega-3 (VITAL) showed that untargeted vitamin D_3_ supplementation did not significantly reduce fracture risk in generally healthy midlife and older adults not selected for vitamin D deficiency, OP or low bone mass [101]. Similarly, vitamin K_2_ as menaquinone-7 (MK-7) has shown variable effects: a three-year trial reported attenuation of age-related decline in BMD and bone strength in healthy postmenopausal women [102], whereas another found no between-group benefit for BMD or microarchitecture in postmenopausal women with osteopenia receiving concurrent calcium and vitamin D [103].

Probiotic findings are also strain- and population-dependent, with *Lactobacillus reuteri* 6475 reducing bone loss in older women with low BMD in one trial [104] but showing no effect on bone loss or bone turnover in early postmenopausal women in another [105]. Overall, current evidence supports a multi-target model in which diet contributes to bone ageing prevention by combining various important factors like dietary patterns like the Mediterranean diet and selected bioactive foods or compounds. Further details on studies are presented in Table 3, with increasing complexity as we go from top to bottom of this table starting from in vitro studies to interventional studies and human trials at the bottom.

### 4.3. Effects of Movement and Exercise on Bone Ageing, Senescence and Inflammaging

Exercise may influence bone ageing through effects on both BM niche and bone tissue itself. The strongest preclinical evidence supports an effect on the age-related shift from osteogenesis toward BMAT. In ageing models, exercise reduces BMAT accumulation through initiation of lipolysis, promotes osteogenesis, and can improve lymphopoiesis, suggesting that movement may help preserve a more osteogenic and less degenerative marrow environment [114,115]. Improvement in lymphopoiesis additionally indicates the maintenance of balance between lymphoid and myeloid cells to prevent myeloid skewing, that often links with several ARDs. Recent work has strengthened this further by showing that exercise suppresses macrophage-derived PCNA clamp-associated factor (PCLAF), thereby reducing BM adipocyte senescence and alleviating skeletal ageing in old mice [116]. Together, these findings suggest that exercise can oppose one of the hallmark features of skeletal ageing: the diversion of the marrow niche away from bone formation and towards adiposity and inflammatory dysfunction [114,116].

Exercise may also attenuate aspects of cellular senescence and inflammaging. Voluntary running has been shown to reduce inflammatory leukocyte production through effects on HSCs [117], while a structured 12-week exercise intervention in older adults lowered circulating and immune cell senescence related markers, including p16, p21, cyclic GMP-AMP synthase (cGAS) and TNF-α [118]. High-intensity and high-impact exercises like combination of heavy resistance training and impact loading activities were found to be effective in maintaining bone density in the spine and femur in postmenopausal women [119]. However, the same set of exercises had little impact when analysed as a meta-study in overweight adults [120]. At the cellular level, mechanical stretch in aged MSCs reduced senescence markers and suppressed SASP while restoring osteogenic potential [121,122]. Thus, the effects of the exercises may vary depending upon the target population and direct evidence that exercise fully reverses established age-related myeloid skewing or bone-specific SASP in vivo remains limited [117,118].

Mechanical loading is also directly relevant to the ageing bone itself. Ageing disrupts the LCN, with fewer canaliculi and reduced interconnectivity, changes that are expected to impair osteocyte mechano-sensing and contribute to fragility [122]. Ageing also suppresses osteocyte perilacunar/canalicular remodelling, reducing collagen integrity and bone quality independently of bone mass alone [123]. In young bone, loading normally suppresses SOST and activates Wnt signalling to stimulate bone formation, but this anabolic response is blunted with age because suppression of SOST and Dickkopf WNT Signalling Pathway Inhibitor 1 (Dkk1) and activation of Wnt1/Wnt7b are not sustained [124].

Accumulation of adipocytes within the BM in advancing age and obesity is reported to impair HSCs, as well as bone regeneration [82]. Exercise-related pathways may partly restore this responsiveness: osteocytic sirtuin 3 (Sirt3) is required for osteocyte dendrite formation and bone gain in response to exercise in aged mice, suggesting a role in maintaining mechano-sensitivity, and activation of connexin 43 (Cx43) hemichannels can rescue part of the diminished anabolic response of aged bone to loading [125,126]. Overall, current evidence suggests that exercise is more convincingly able to preserve skeletal homeostasis during ageing than to fully reverse established structural degeneration.

### 4.4. Other Existing and Emerging Strategies

Beyond diet and exercise, several clinically relevant strategies can help promote healthy skeletal ageing and reduce the burden of OP, frailty, and fractures. A key approach is the implementation of frailty and sarcopenia programmes, incorporating muscle-strengthening, balance training, mobility assessment, and early identification of declining physical function [127]. Preserving muscle mass and strength supports mechanical loading of bone and reduces fracture risk. Fall-prevention programmes are equally important and may include home hazard assessment, vision correction, footwear optimisation, medication review, and balance-focused rehabilitation [128]. As many osteoporotic fractures result from falls, reducing fall risk is a major anti-ageing strategy for bone health.

Control of inflammaging may help limit age-related bone loss by reducing osteoclast activation and preserving osteoblast function. Management of inflammatory diseases and optimisation of lifestyle and metabolic health are therefore important. Optimising endocrine and metabolic comorbidities, including vitamin D deficiency, diabetes, thyroid disease, chronic kidney disease, and hypogonadism, can improve bone remodelling and reduce fracture risk.

Senolytics and senomorphics have emerged as promising strategies to target the cellular senescence that contributes to skeletal ageing. Over the past decade, preclinical studies have shown that clearance of senescent cells using the senolytic combination dasatinib plus quercetin (D + Q) improves bone formation, reduces bone resorption, and enhances bone microarchitecture in aged mice [129]. Similarly, the flavonoid fisetin has demonstrated senolytic activity and beneficial effects on age-related tissue dysfunction, including bone. Senomorphic approaches aim to suppress SASP without killing cells; agents targeting JAK/STAT, mTOR, and inflammatory signalling pathways have been shown to reduce skeletal inflammation and bone loss in animal models. These interventions are thought to preserve osteoblast function, improve osteocyte health, and reduce osteoclast-driven bone resorption. While early human senolytic trials have established feasibility and safety in age-related conditions, definitive clinical trials specifically targeting bone ageing and OP remain ongoing and represent an important future research priority.

## 5. Conclusions: Challenges, Limitations and Future Directions

### 5.1. Challenges and Limitations

Using bone as a biomarker for ageing and cellular senescence, however, represents several challenges. As we have discussed above, it is a highly dynamic tissue influenced by mechanical loading, several pathways, nutrition and exercises which can confound ageing-specific signals. Cellular heterogeneity within the BM including MSCs, HSCs, and immune cells complicate our interpretation of senescence markers. Additionally, sampling the bone is invasive and perhaps, the most limiting challenge in the field. Thus, sampling often remains limited to specific anatomical sites, reducing representativeness and the chances of truly comparing a healthy donor of any age to another disease. Moreover, ageing is heterogenous even within the bone, with osteocytes within the cortical bone being the longest living cells, in comparison with the same cells from spongy bone that renew relatively faster.

Thus, to truly study bone ageing, the investigations need to be spaced locally. While spatial investigation in bone would be ideal, it is not always feasible and thus blood is used to investigate ageing. Evidence suggests that osteocytes that were long considered passive are actually the main mechano-sensors of the bone, form a highly connected communication network and are central to health, ageing and diseases; especially considering their life-span. Osteocytes may well be the cells whose survival mechanisms will be key targets in health research as accelerated cell death leads to bone fragility [130].

Senescence markers such as p16^INK4a^ and SASP factors currently lack specificity, standardisation and reflect inflammation, rather than true ageing or inflammaging. Finally, donor variability and cell-culture-based in vitro ageing that sets in due to population doublings further limit the reliability of distinguishing in vitro ageing from physiological in vivo ageing, and of using bone-derived cells as a universal biomarker of ageing [131]. Genetic and cellular alterations that interfere with bone production, resorption, and tissue homeostasis, including the buildup of senescent cells, cause bone ageing. These mechanisms are regulated by important genes like *SIRT1*, *RUNX2*, *FOXO3*, and *TERT*. In order to maintain bone health and prevent diseases like OP, integrating genomes with functional investigations may identify causative pathways and direct interventions like senolytic or gene-targeted medications. Additionally, pathways of DNA damage, ROS and senescence that play a key role in bone ageing [132] need an optimised platform that replicates the BM niche closely.

Thus, the challenge of the lack of relevant biomimetic model consistently remains. This challenge of limited access to physiologically relevant models truly reflecting the healthy bone provides us with a unique opportunity to fabricate novel platforms using 3D, microfluidic chip or organ-on-chip (OOC), specifically for the bone to reflect both healthy (young and old) as well as diseases in the bone. Interest specifically in this area is increasing with the use of natural materials for biomimetic scaffolds [133], combination of cutting-edge technologies like next generation sequencing (NGS) with bone organoids to provide information about pathways and mechanisms for MSK ARDs [134], that we have not had access to before, and OOCs, providing a step towards precision medicine [135,136].

### 5.2. Translational Significance, Roadmap and Future Directions

Importantly, bone ageing is increasingly recognised as a spatially structured phenomenon, with distinct microanatomical niches (e.g., endosteal and perivascular regions) exhibiting divergent cellular composition and signalling dynamics [137]. Emerging spatial transcriptomic approaches have begun to address these limitations by enabling in situ profiling of gene expression within intact tissue architecture, revealing gradients and localised hotspots of biological ageing. However, adoption in bone research remains constrained by technical challenges, including tissue decalcification, RNA preservation, and data integration across scales.

Short-term goals should focus on establishing standardised, reproducible bone senescence biomarkers that can be integrated into clinical practice alongside DXA. This includes validating circulating markers (e.g., P1NP, CTX, SOST, RANKL/OPG), imaging measures (TBS, cortical porosity, HR-pQCT), and cellular senescence signatures to identify individuals with accelerated skeletal ageing before diseases develop. For mid-term goals, aims to develop composite “bone age” scores that integrate molecular, imaging, functional, and fracture-risk data will provide a clearer pathway into clinics. These tools should enable stratification of biological versus chronological ageing, monitor responses to exercise, nutrition, and pharmacological interventions, and identify links between bone ageing, sarcopenia, vascular calcification, and frailty. Long-term goals should position bone as a systemic biomarker of healthy ageing and senescence. Bone senescence testing could become part of precision-geroscience, helping predict multimorbidity, disability, cardiovascular (CV) disease and longevity. Additionally, integration of bone biomarkers with muscle, vascular, immune, and gut-health markers may provide a comprehensive assessment of biological ageing and guide targeted senotherapeutic interventions.

**Figure 4:**
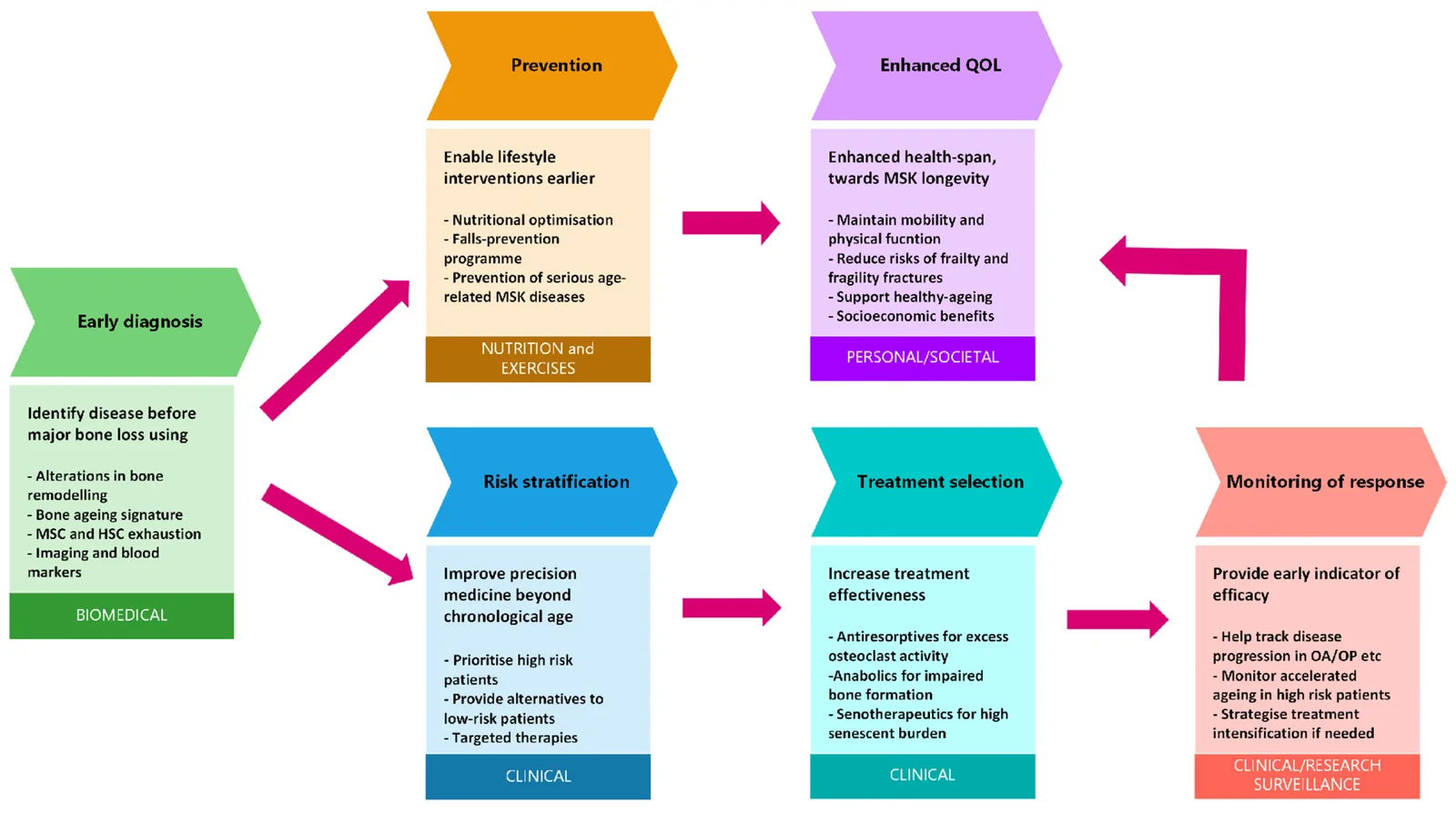
Translational significance of tracing bone senescence biomarkers across the clinical continuum of healthy ageing.

Identifying robust bone ageing biomarkers could transform the management of skeletal health across the prevention-to-treatment pathway (Figure 4). The figure illustrates how bone senescence testing could support a precision-medicine approach to MSK ageing. At the early diagnosis stage, bone-specific imaging, blood biomarkers, and signatures of bone remodelling, MSC and HSC exhaustion may identify age-related skeletal decline before substantial bone loss or clinical disease develops. This enables prevention through earlier lifestyle interventions, including nutritional optimisation, exercise programmes, and fall-prevention strategies. Biomarkers can then inform risk stratification, distinguishing biological from chronological ageing and identifying individuals at greatest risk of OP, frailty, MSK diseases and fracture. In the clinical setting, biomarker profiles may guide treatment selection, supporting targeted use of antiresorptive, anabolic, or emerging senotherapeutic approaches. Ongoing monitoring of response provides an early indication of treatment efficacy and disease progression, enabling timely adjustment of management strategies, spanning all diseases impacted by bone ageing.

The future of using bone as a biomarker of ageing thus lies in integrating advanced technologies such as single-cell omics, spatial transcriptomics and AI-driven imaging to capture cell-specific and niche-level changes [138]. However, these should still be in combination with blood-based tests and DXA and μCT for enhanced accuracy. In 2023, the Aging China Biomarkers Consortium (ABC) provided a consensus on recommended methodologies for biomarkers in skeletal ageing. They suggested classifying these markers based on imaging (change in bone and cartilage structure and metabolism), functional (limb mobility, posture control) and body fluids (including MMPs, IL-6 and even gut microbiota) [139]. More importantly, there is a growing body of evidence indicating links between bone ageing and CV health, referred to as the bone–vascular axis [140]. Studies have indicated that a BMD of over 0.741 gm/cm^2^ predicts OP risk [141] and increased BMD is associated with higher CV risk and increased calcification with age [142]. Future should directly investigate the impact of CV health with bone ageing, and a deeper quest in the ageing gut–bone axis will be crucial in expanding our knowledge in the field for enhancing health-span in the elderly.

These approaches will improve predictive accuracy by linking molecular signatures (e.g., senescence markers, epigenetic clocks) with numerical as well as functional age-related changes. In precision medicine, bone-derived data could enable individualised ageing profiles and targeted interventions. Longitudinal, multi-omics datasets will enhance the ability to distinguish normal ageing from pathology. Ultimately, combining cellular, structural and molecular indicators will allow bone to more reliably reflect systemic, age-related changes and biological age. Most of all, longitudinal studies that investigate the effect of at least a combination of two approaches from the mix of the three pronged anti-ageing strategies (for example, Biologics + exercise or Pharmaceutical approaches + exercise or Pharmaceutical approaches + diet) or all the three strategies will be essential in truly exploring the longevity of bones and the MSK system and encourage research for biologics like PRP [143] and anti-ageing based materials for bone regeneration [144].

## Figures and Tables

**Table 1:** Age-related changes in functions of BM stem cells, reported in the last 5 years.

Stem Cell Type	Function	Model	Age-Related Changes	Reference
**MSCs**	Proliferation and regenerative capacity	Human BM-MSCs from young (<18 years old, *n* = 28) and old (>55 years old, *n* = 20) donors	Old donor MSCs decreased colony-forming ability, impaired cell-cycle progression, and accumulation of senescence markers (e.g., SA-β-gal, ROS)	[53]
	Differentiation	Human BM-MSCs from young (30 and 45 years old) and old (60 and 80 years old) donors	Older donor BM-MSCs displayed impaired osteogenic capacity, reduced mineralisation and lower expression of osteogenic markers (e.g., Runx2, osteopontin)	[57]
	Supporting haematopoiesis	CD146^+^ BM-MSCs from middle-aged (≤66 years old, *n* = 13) and old (>75 years old, *n* = 16); co-culture with CD146^+^ MSC and CD34^+^ HSCs	MSC from older donors fail to support HSPC migration and mobilisation effectively, even under stimulatory conditions	[59]
**HSCs**	Myeloid skewing	CD34+ HSCs from middle-aged (50–64 years old) and older adults (65–92 years old)	Ageing increases maturing myeloid cell density surrounding adipocytes, contributing to age-related risk of myeloid malignancies.	[15]
	Self-renewal	*n* = 299 patients who underwent peripheral blood haploidentical haematopoietic cell transplantation	Aged HSCs exhibit reduced capacity to remain in a quiescent, non-cycling state, leading to exhaustion and haematopoietic dysfunction. Thus, increasing donor age is associated with worse outcome in HSC transplantation	[60]
	Clonal expansion and heterogeneity	3579 genomes from single cell-derived colonies of HSCs from *n* = 10 human subjects (0 to 81 years old)	Rapidly decreasing clonal diversity was outlined as a universal feature of haematopoiesis in aged humans	[61]

Table footnote: MSCs: mesenchymal stem/stromal cells; HSCs: haematopoietic stem cells; SA-β-gal: senescence-associated beta-galactosidase; ROS: reactive oxygen species.

**Table 2:** Current biomarkers of bone ageing.

Biomarker	Biological Role	Relevance in Bone Ageing
Bone Mineral Density (BMD)	Measures bone mineral content and overall skeletal mass using DXA.	Gold-standard clinical marker of skeletal ageing; declining BMD is associated with OP, fragility fractures, and age-related bone loss. However, BMD does not fully capture bone quality or microarchitecture.
Trabecular Bone Score (TBS)	DXA-derived measure reflecting trabecular microarchitecture.	Provides information on bone quality independent of BMD and may identify age-related deterioration in skeletal structure before substantial bone loss occurs.
P1NP (Procollagen Type I N-terminal Propeptide)	Marker of osteoblast activity and new collagen formation during bone formation.	Reduced levels may indicate impaired bone formation associated with ageing and osteoblast dysfunction; widely recommended as a reference bone formation marker.
CTX (C-terminal Telopeptide of Type I Collagen)	Marker of osteoclast-mediated bone resorption.	Elevated CTX reflects increased bone turnover and accelerated skeletal ageing, particularly after menopause. Commonly used to monitor antiresorptive therapies.
Sclerostin (SOST)	Osteocyte-derived inhibitor of the Wnt/β-catenin pathway.	Levels generally increase with ageing and suppress bone formation, contributing to reduced osteoblast activity and impaired skeletal adaptation to mechanical loading.
RANKL	Stimulates osteoclast differentiation and activation.	Increased RANKL signalling shifts bone remodelling toward resorption, a hallmark of age-related bone loss and OP.
Osteoprotegerin (OPG)	Decoy receptor for RANKL that inhibits osteoclastogenesis.	Altered RANKL/OPG balance is associated with skeletal ageing and may also link bone loss with vascular calcification.
FGF23 (Fibroblast Growth Factor-23)	Regulates phosphate and vitamin D metabolism.	Dysregulation contributes to mineral metabolism abnormalities observed in ageing, OP, chronic kidney disease and vascular calcification.
Cortical Porosity	Structural imaging biomarker reflecting cortical bone deterioration.	Increases with age and contributes substantially to reduced bone strength and fracture susceptibility, even when BMD changes are modest.
Trabecular Volumetric BMD (vBMD)	Measures density of trabecular compartments using QCT/pQCT.	Sensitive indicator of age-related microarchitectural deterioration and responsiveness to mechanical loading.
Senescent Osteocyte Burden (p16INK4a, p21, SASP markers)	Reflects accumulation of senescent bone cells and secretion of pro-inflammatory SASP factors.	Emerging biomarker of biological bone age; linked to impaired mechano-transduction, reduced bone formation, inflammaging, and skeletal fragility.
Mesenchymal Stem Cell (MSC) Osteogenic Capacity	Indicates ability of progenitor cells to differentiate into osteoblasts.	Ageing reduces MSC osteogenic potential and promotes adipogenesis, contributing to impaired bone regeneration and OP.
Global Cardiac Calcium Score (GCCS) (cross-system ageing biomarker)	Measures valvular and cardiac calcification burden.	Not a bone biomarker per se, but increasingly associated with low BMD, fragility fractures, sarcopenia, and skeletal ageing through the bone–vascular axis.
Gut Microbiome-Derived Metabolites (e.g., SCFAs)	Regulate immune signalling, intestinal permeability, calcium absorption, and bone remodelling.	Emerging biomarkers of the gut–bone axis; age-related dysbiosis may promote inflammaging and osteoporotic bone loss.

**Table 3:** Summary of studies investigating the effects of food, nutrition and diet on different conditions of the MSK system.

Study Type	Conditions, Diseases, and Models Investigated	Diet-Related: Food, Supplements, Dietary Patterns, Cuisines	Key Findings	Reference
In vitro	Young and senescent human bone marrow-derived mesenchymal stromal cells	Orthosilicic acid, vitamin K_2_, curcumin, polydatin and quercetin combination	Increased osteogenic markers including ALP/COL1A1 and reduced SASP-related IL-1β, IL-6, IL-8 and MCP-1, suggesting pro-osteogenic and anti-inflammatory activity in senescent bone-forming progenitors	[92]
In vitro/in vivo	RANKL-induced osteoclastogenesis and ovariectomy-induced OP	4-hydroxyphenylacetic acid, (microbial polyphenol metabolite)	Reduced ROS, NF-κB/MAPK activation and osteoclast genes including NFATc1, CTSK, MMP9 and Acp5. Micro-CT/histology indicated protection from OVX bone loss	[94]
In vivo	Oestrogen-deficiency bone loss in C57BL/6 mice	Dried plum, including polyphenol and carbohydrate fractions	Dried plum components contributed to osteoprotection and prebiotic activity, implicating gut-derived mechanisms in postmenopausal bone ageing	[93]
In vivo	Ageing bone exposed to short-term high-fat diet in adult and aged C57BL/6JN mice	High-fat diet (45% dietary fats)	Exacerbated loss of bone matrix quality and fracture resistance; affected osteoclast/adipocyte abundance and osteocyte viability	[106]
In vivo	Active vitamin D insufficiency and skeletal ageing between Male wild-type (WT) and Cyp27b1 haplo insufficient mice	Active vitamin D insufficiency; rescue with 1,25-(OH)2D_3_, antioxidant or p16 inhibition	Linked vitamin D insufficiency to ROS, DNA damage, p16-mediated senescence/SASP and bone loss	[107]
In vivo	Oestrogen-deficiency OP in ovariectomised mouse model	*Bifidobacterium* strains (probiotics)	Improved BMD, bone volume and trabecular number; suppressed inflammation and osteoclast generation through intestinal immune modulation	[108]
Ex vivo human sub-study (Parent trial *n* = 235)	Postmenopausal women from the Prune Study	50–100 g/day prunes	Attenuated pro-inflammatory cytokine secretion and altered monocyte activation, linking prune intake to lower immune inflammaging signals relevant to bone resorption	[100]
Observational study (*n* = 3995)	Community-dwelling older Chinese adults	Dietary inflammatory potential/Dietary Inflammatory Index	Higher inflammatory dietary potential associated with adverse musculoskeletal health measures, supporting a diet-inflammaging-bone link	[109]
Observational study (*n* = 927,130)	Osteoporotic fracture risk in a large claims-based cohort	Meal timing: breakfast skipping and late dinner	Breakfast skipping and late dinner were associated with increased osteoporotic fracture risk, suggesting chrononutrition may influence skeletal ageing risk	[110]
Human trial/intervention (*n* = 924)	Older adults with overweight/obesity and metabolic syndrome	Energy-reduced Mediterranean diet plus physical activity and behavioural support	Mitigated BMD decline over 3 years, especially lumbar-spine BMD in women	[111]
Human trial (*n* = 239)	Early postmenopausal bone loss	*Limosilactobacillus reuteri* 6475 (probiotics)	Did not prevent bone loss or alter bone turnover over 2 years, highlighting heterogeneity in probiotic bone effects	[105]
Human trial (*n* = 40)	Postmenopausal women with osteopenia	Multispecies probiotic supplementation	Reduced serum CTX, suggesting a short-term antiresorptive effect	[112]
Human trial (*n* = 235)	Postmenopausal women	50 g/day or 100 g/day prunes for 12 months	Preserved total hip BMD; 50 g/day was the most feasible dose and prevented hip BMD loss	[95]
Human trial (*n* = 25,871)	Generally healthy midlife and older adults	Vitamin D_3_, 2000 IU/day	Did not significantly reduce total, nonvertebral or hip fractures, suggesting limited benefit of untargeted supplementation in replete populations	[101]
Intervention study (*n* = 7195)	Older adults in residential aged-care facilities	Milk, yoghurt and cheese to increase calcium and protein intake	Reduced all fractures, hip fractures and falls without changing mortality, supporting food-based protein/calcium sufficiency	[113]

## Data Availability

No new data were created or analysed in this study. Data sharing is not applicable to this article.

## Abbreviations

The following abbreviations are used in this manuscript:

3D	Three-dimensional
ARDs	Age-related diseases
BM	Bone Marrow
BMAT	Bone marrow adipose tissue
BMD	Bone mineral density
BTM	Bone turnover marker
cGAS	cyclic GMP-AMP synthase
CTX	C-terminal telopeptide
CV	Cardiovascular
Cx43	Connexin 43
DKK1	Dickkopf-1
DNA	Deoxyribonucleic acid
DXA	Dual-energy X-ray absorptiometry
ECM	Extracellular matrix
HSCs	Haematopoietic stem cells
FGF23	Fibroblast growth factor 23
FOXO3	ForkHead box O3
FRAX	Fracture risk assessment tool
HR-pQCT	High-resolution peripheral QCT
IL-6	Interleukin 6
IL-8	Interleukin 8
LCN	Lacuno-canalicular communication
LRP5	Low-density lipoprotein receptor-related protein 5
MK-7	menaquinone-7
MSCs	Mesenchymal stem/stromal cells
MSK	Musculoskeletal
mTOR	Mechanistic target of Rapamycin
NGS	Next-generation sequencing
OA	Osteoarthritis
OOCs	Organ-on-chip
OP	Osteoporosis
OPG	Osteoprotegerin
QOL	Quality of Life
P1NP	Procollagen type 1N propeptide
PCLAF	PCNA clamp-associated factor
PDGF	Platelet derived growth factor
PPAR-γ	Peroxisome Proliferator-Activated Receptor gamma
PRP	Platelet-rich plasma
RA	Rheumatoid arthritis
RANK	Receptor activator of nuclear kappa beta
RANKL	Receptor activator of nuclear kappa beta ligand
RNA	Ribonucleic acid
ROS	Reactive oxygen species
RUNX2	Runt related transcription factor 2
SA-β-gal	Senescence-associated beta-galactosidase
SASP	Senescence-associated secretory phenotype
Sirtuin 1	Sirt 1
Sirtuin 3	Sirt 3
SNC	Shell nacre cement
SOST	Sclerostin
STNG	Stimulator of interferon genes
TBS	Trabecular bone score
TNF-α	Tumour necrosis factor-alpha
TERT	Telomerase reverse transcriptase
TGF-β	Transforming growth factor-beta
VEGF	Vascular endothelial growth factor
Wnt	Wingless related integration site
